# Stranger than Fiction: Costs and Benefits of Everyday Confabulation

**DOI:** 10.1007/s13164-017-0367-y

**Published:** 2017-10-26

**Authors:** Lisa Bortolotti

**Affiliations:** University of Birmingham, Birmingham, UK

## Abstract

In this paper I discuss the costs and benefits of confabulation, focusing on the type of confabulation people engage in when they offer explanations for their attitudes and choices. What makes confabulation *costly*? In the philosophical literature confabulation is thought to undermine claims to self-knowledge. I argue that when people confabulate they do not necessarily fail at mental-state self-attributions, but offer ill-grounded explanations which often lead to the adoption of other ill-grounded beliefs. What, if anything, makes confabulation *beneficial*? As people are unaware of the information that would make their explanations accurate, they are not typically in a position to acknowledge their ignorance or provide better-grounded explanations for their attitudes and choices. In such cases, confabulating can have some advantages over offering no explanation because it makes a distinctive contribution to people’s sense of themselves as competent and largely coherent agents. This role of ill-grounded explanations could not be as easily played by better-grounded explanations should these be available. In the end, I speculate about the implications of this conclusion for attempting to eliminate or reduce confabulation.

## Introduction

There are several forms of behaviour that are described as instances of confabulation in the empirical literature. ‘Confabulation’ comes from the Latin *fabula*, which means ‘story’. A story can be a faithful representation of reality that aims at accuracy, such as a historical account; or a piece of fiction that does not aim at accuracy, such as a fairy-tale. People confabulate when they tell a story that is not backed up by the relevant evidence, although they genuinely regard it as a true story. Thus, in terms of its relationship with evidence, the story has the same status as a piece of fiction, but in terms of people’s intentions in telling the story, it is offered as a faithful representation of reality. Indeed, people do not *intentionally* confabulate. If they report something that they know to be untrue or ill-grounded, then they do not confabulate, but engage in a form of lying or deceit.

In this paper, I am interested in the ill-grounded explanations people offer for their attitudes and choices when they are not aware of some of the key factors causally responsible for their behaviour. In the philosophical literature the costs of this form of confabulation have been discussed at length. In particular, confabulation is construed as a challenge to mental-state self-ascriptions and first-person authority (e.g., Carruthers 2005; Lawlor 2003; Scaife 2014). The benefits of confabulation have not been investigated in detail in the philosophical literature (but see Bortolotti and Cox 2009; Strijbos and de Bruin 2015; Coltheart 2017) and, in particular, it has not been asked whether confabulation can have *epistemic* benefits. This leaves us with a potentially one-sided view of the epistemic status of confabulation.

Here I propose a new account of what is epistemically problematic about confabulation and make room for the view that confabulation has psychological and epistemic benefits that deserve careful consideration. These are not benefits that agents *intend* to gain by confabulating, but aspects of confabulation that make a positive epistemic contribution independent of the agent’s intentions. In Sections 2, 3, and 4, I introduce and articulate the notion of confabulation. My main focus is the phenomenon of offering ill-grounded explanations for everyday choices, but the same features of confabulation I identify in that context can also be found in explanations of moral judgements and hiring decisions. My thesis is that confabulation is a type of ill-grounded explanation for attitudes and choices that is offered when the causal processes responsible for such attitudes and choices are opaque to introspection or otherwise difficult to access.

In Sections 5, 6 and 7, I turn to the costs and benefits of confabulation. Confabulation does not necessarily involve a breakdown of self-knowledge. People who confabulate know what their attitudes and choices are, but do not have access to key information about the formation of those attitudes and choices. When they provide an ill-grounded explanation rather than acknowledging ignorance, they often end up adopting further ill-grounded beliefs. This represents a significant epistemic cost. However, confabulating may support people’s sense of themselves as competent and largely coherent agents to a greater extent than acknowledging ignorance or offering better-grounded explanations for attitudes and choices, and it can also have some epistemic benefits. Psychological evidence suggests that, when people develop a sense of themselves as competent and largely coherent agents, then they interact with their environment in a way that is more conducive to the acquisition, retention, and use of relevant information, and they become more efficacious and resilient at pursuing their goals, including their epistemic goals.

In Section 8, I consider some objections to the thesis that confabulation has epistemic benefits, and I reflect on some implications of my view for interventions aimed at reducing confabulation, both in social exchanges and in personal reflections about attitudes and choices.

## What is Confabulation?

Clinically, confabulation is a symptom of neuropsychological disorders featuring memory impairments. Henry, who has memory impairments due to frontal damage, reports correctly that he is married and that he has four children. When he is asked how long he has been married, he replies incorrectly “4 months”, instead of 30 years (*primary confabulation*). When he is asked how it is that he has four children after only 4 months of marriage, he claims that his children were adopted, even though they are in fact his natural children (*secondary confabulation*).^1^ In the former instance, Henry makes an inaccurate claim about his past; in the latter instance, Henry makes a further inaccurate claim in order to defend his initial report from a challenge. There is no reliable autobiographical information on which Henry can base his answers given his memory impairment. Henry distorts reality in significant ways (his four children were *not* adopted), but his answers to the questioning are sincere and they are not offered with the intention to deceive. Henry reports what he believes to be the case, filling the gaps in the knowledge of his past with hypotheses that are plausible given what he knows.

Non-clinical confabulation captures a much more widespread phenomenon which is defined in terms of the *epistemic features* of the claims that people produce. Researchers do not agree about which epistemic costs are shared by different instances of confabulation (Bortolotti and Cox 2009). According to some influential proposals, confabulation involves *inaccurate narratives* or *false beliefs* that are also *resistant to counter-evidence* (Berrios 2000, page 348; Turnbull et al. 2004, page 6). Such proposals make inaccuracy or falsehood necessary for confabulation. Alternative accounts identify the main cost of confabulation with producing narratives or adopting beliefs that are *not based on the relevant evidence*. For instance, confabulation has been defined as an *ill-grounded claim* people make when they do not realise that their claim is ill-grounded (Hirsten 2005), or an *unsubstantiated claim* people make in response to a question that they cannot answer because the relevant information is not known or accessible to them (Turner and Coltheart 2010).

The definitions of confabulation based on ill-groundedness or lack of evidential support are preferable to those based on inaccuracy or falsehood, as someone lacking access to the relevant evidence may still produce an accurate narrative or a true claim *by accident*, without relying on the evidence supporting the claim. That said, most cases of confabulation, including those I will discuss in this paper, do result in inaccurate narratives or false claims.

## Confabulating about Consumer Choice

People often confabulate when they are asked about their attitudes or choices.

When a person does not know or does not have access to the answer to a question addressed to that person (typically the question may be a request for explanation of why the person behaved in a certain way, or else a question asking why the person holds a particular belief), but when asked the question responds by offering an answer to it rather than saying ‘I don’t know’, and if this is done with no intention to deceive the questioner, then that response counts as confabulation (Coltheart and Turner 2009, page 180).

In their now classic study, Richard Nisbett and Tim Wilson wanted to investigate the extent to which people are aware of their mental processes when they are asked to give reasons for their choices (Nisbett and Wilson 1977). Research participants were asked to choose some items as part of a consumer survey. Some participants were asked to choose between four nightgowns which were different from one another. Other participants were asked to choose between four pairs of nylon stockings which were identical. Then, all participants were asked why they made their choices. The experimenters found that participants’ choices were very heavily influenced by the position of the items, and the item that was most on their right was the one they systematically preferred.^2^ But when people offered reasons for their choices, they did not mention the position of the chosen items as a factor determining or influencing their choices. Rather, they mentioned features of the items, such as softness or colour, even when the items they chose from differed only in their position.

What happens in the Nisbett and Wilson study? There is some controversy about the best interpretation of the study.^3^ The authors argue that, when participants are asked to explain their choices, they have no access to the mental processes responsible for their choices because such processes are characterised by priming effects that are opaque to introspection. Instead, participants provide an explanation that is plausible given their background beliefs about what makes items such as nightgowns or stockings preferable. As a result, their explanations are not grounded on the evidence relevant to the processes responsible for their making those particular choices.

Imagine that three research participants are asked, “Why did you choose this pair of nylon stockings?” just after they have chosen one pair out of four identical pairs. Also imagine that they all claim that they chose that pair because it was the *most brightly coloured*, but they arrive at their answers differently. Are their claims an instance of confabulation? What is wrong with their claims?

Sylvia chooses the rightmost pair of stockings because she believes that it is the brightest. She explains that she chose it because it is the brightest. The explanation she offers for her choice is accurate, although her belief about the chosen pair of stockings being the brightest is false. In this first scenario, Sylvia does not engage in confabulation, because she offers an explanation for her choice that is well-grounded. But her choice is based on a false belief.

Roberto chooses the rightmost pair of stockings because of position effects. When asked to explain his choice, he answers that he chose that pair because it was the brightest. As Roberto does not mention the role of position effects in his choice, his explanation is ill-grounded. In this case, Roberto confabulates. Not only does he offer an ill-grounded explanation, but, as a result of confabulating, he also forms the belief that the pair of stockings he chose is the brightest, and that belief is false. In this second scenario, we find the interpretation of the experimental results defended by Nisbett and Wilson. Participants who are asked for an explanation of their choices produce an ill-grounded causal claim due to their ignorance of the mental processes underlying their choices.

Swati chooses the rightmost pair of stockings because of position effects. She interprets the experimenter’s question about her choice as a request for a justification. Presumably, there is no good reason for Swati’s choice as the stockings to choose from were identical (unless we believe that we are in a situation in which the position of an item is a good reason to choose it). When asked to justify her choice, Swati says that the pair of stockings she chose is the brightest. Her justification latches onto generally plausible reasons for choosing stockings and other similar items. However, as a justification for her particular choice of the rightmost pair of stockings within a set of four identical pairs of stockings, her answer is epistemically problematic. In this third scenario, Swati’s answer is not supposed to disclose the causal processes leading to her choice, but to highlight what good reasons there are for that choice, whether or not those reasons did motivate her in making the choice (Sandis 2015). The problem is that the reason she mentions as a justifying reason for her particular choice does not match the features of the situation.

Independent of the interpretation of the experimental results which we favour, there are significant epistemic faults in what people say about their choices. In Sylvia’s case, we have a well-grounded and accurate explanation based on a false belief. Sylvia gets the world wrong (the chosen pair of stocking is not the brightest), but she accurately identifies the reasons for her choice. In Roberto’s case, we have a case of confabulation resulting in the adoption of a false belief, that the chosen stockings are the brightest. Roberto is not aware of the factors determining or influencing his choice and he provides an ill-grounded and inaccurate explanation for his choice. In Swati’s case, she interprets the task as a request for justification and thus she does not even attempt to identify the causal processes leading up to her original choice. The justification Swati offers for her choice is epistemically problematic and, in searching for a justification, she ends up adopting a false belief, that the chosen stockings are the brightest*.*

The possibility that the cases of Sylvia and Swati illustrate what occurs some of the time when people are asked for reasons should not be ruled out. In the Nisbett and Wilson study, for instance, it is possible that position effects generate a perceptual salience which manifests as brightness for some participants and as softness for other participants. This may give rise to the situation described in Sylvia’s case. Also, it is possible that people interpret the question “Why did you choose this pair of stockings?” as a request for justification rather than as a request for explanation, due to the question being ambiguous. That said, with Nisbett and Wilson, I will assume here that the most plausible interpretation of the behaviour of the participants in the study is that they *offer an ill-grounded explanation* as in Roberto’s case.

Some of my considerations in the rest of the paper, however, can also apply to different interpretations of the way in which people account for their attitudes and choices. Notice that Sylvia explains her choice as based on a belief that she indeed has, the belief that the item most on the right is the brightest, but she may not realise that her belief is influenced by priming effects and that the belief could be different if the position of the item changed. So, the concerns one might have about the explanation in Roberto’s case, that it may indicate a limitation in people’s knowledge about how their attitudes are formed or their choices are made, may apply to Sylvia’s initial belief too. Similarly, it is true that explaining and justifying are distinct enterprises with different success conditions, but in rational decision making the reasons that motivate people to make a choice should at least partially reflect what people take to be good reasons for that choice. The reasons the participants offered for their choices are not likely to be either motivating reasons for those choices or suitable reasons in support of those particular choices. Which means that in Swati’s case just as in Roberto’s case some epistemic failure is at play.

One interesting feature about the Nisbett and Wilson study is that the participants’ story about why they made their choices rings more true than the accurate explanation of their behaviour, especially on the background of the assumption that, generally, humans are rational agents and competent decision makers. Why should people be influenced by the position of the items in the context of consumer choice? Isn’t it more plausible that the pair of stockings was chosen because of its texture than because of its position? As we saw, Nisbett and Wilson argue that, not having access to the mental processes responsible for their choices, people offer an explanation of their choices based on plausibility considerations. Thus, this form of non-clinical confabulation applies to cases where *truth is stranger than fiction*:

There is a class of influential factors to which we should be particularly blind. That class may be described as the mechanics of judgment factors—for example, serial order effects, position effects, contrast effects, and many types of anchoring effects. Such factors should seem particularly implausible as reasons for liking or disliking an object, or for estimating its magnitude on some dimension as high or low. *Indeed, it seems outrageous that such a judgment as one concerning the quality of a nightgown might be affected by its position in a series* (Nisbett and Wilson 1977, page 252, my emphasis).

## Confabulating about Moral Judgements and Hiring Decisions

In this section, I will consider two further cases that can be accounted for in terms of an ill-grounded and inaccurate claim resulting in the adoption of further ill-grounded beliefs. The examples show that confabulation is not confined to consumer choice, but can also be detected when people are asked about their moral judgements and their hiring decisions.

In a study by Jonathan Haidt people are presented with the following scenario:

Julie and Mark are brother and sister. They are travelling together in France on summer vacation from college. One night they are staying alone in a cabin near the beach. They decide that it would be interesting and fun if they tried making love. At very least it would be a new experience for each of them. Julie was already taking birth control pills, but Mark uses a condom too, just to be safe. They both enjoy making love, but they decide not to do it again. They keep that night as a special secret, which makes them feel even closer to each other (Haidt 2001, page 814).

Participants are asked whether it was wrong for the siblings to make love, and why. Most people answer that it was wrong for Julie and Mark to make love, but they struggle to come up with reasons for their judgement. Haidt calls this phenomenon “moral dumbfounding”. When probed, some argue that incest is likely to have negative psychological consequences for Julie and Mark, ruin their relationship, and give rise to inbreeding. But the scenario was constructed in such a way as to rule out these possibilities. We know from the brief description of the events that Julie and Mark go on to have a good relationship, and that they use two forms of birth control.

Haidt’s interpretation is that people do not know the psychological mechanisms responsible for their moral judgements. According to the view he defends, *social intuitionism*, what causes people’s moral judgements is a socially conditioned, basic emotional reaction (i.e., disgust towards incest) to which they have no introspective access. When people are asked about their moral judgement, they offer a plausible explanation for rejecting incest as a morally objectionable practice (e.g., that it may have bad consequences for the people involved). But the claim people offer is ill-grounded as an explanation of how they formed their judgement and does not fit with the evidence available to them. In providing an explanation, they commit to claims that lack support and do not fit the specifics of the scenario, such as “Julie and Mark may have a baby”, or “Their relationship is likely to suffer”.

Just as the results of the Nisbett and Wilson study, the results of Haidt’s study could be interpreted differently. For instance, participants may offer an explanation that involves no confabulation and is based on a false or poorly supported belief (“The siblings may have a baby”); or they may offer a justification for their judgement as opposed to an explanation of it. In the latter case, the reason they offer is epistemically problematic as a justification of the moral judgement about incest in the specific scenario because, say, there is no evidence suggesting that Julie and Mark are likely to have a baby as a result of their sexual encounter.

One could argue that according to some views of moral judgement a practice like incest can be judged to be morally wrong without further explanation, because it breaks a rule of moral conduct and needs not be evaluated further on the basis of the consequences for the people involved. Thus, the objection would go, a dumbfounding response is not evidence of confabulation but an appropriate response to the request for explanation. This is a fair point that raises a more general issue: when is it that we should be able to offer an explanation for our choices and attitudes? That said, there is additional evidence suggesting that reactions of disgust affect moral judgement without the person realising it, and such evidence is not vulnerable to the same objection, because the source of the reaction of disgust is not the human practice to be evaluated, but the environment in which the judgement is made.

For instance, in the study by Simone Schnall et al. (2008), discussed in some detail by Strijbos and de Bruin (2015), reactions of disgust caused by elements *external* to the scenario to be evaluated (e.g., a bad smell or a dirty desk in the room where the evaluation is made) increase the severity of the moral judgements. Research participants are not aware of the effects of the ‘disgusting stimuli’ on their judgements and this supports the idea that there are causal factors affecting aspects of attitude formation that people are not aware of and that they are not likely to acknowledge.

Another context where we observe non-clinical confabulation is that of hiring decisions. There is copious evidence suggesting that decision makers are biased by other people’s gender, ethnic background, and sexual preferences in selecting job candidates based on CVs, and also by people’s appearance (especially weight and height in relation to gender) in assessing job candidates’ interviews.^4^ These biases are often implicit, which means that decision makers may not be aware of all the factors that impact on their choices.^5^

For instance, consider the director of a company who has just been involved in a hiring process. She says that she chose Tim over Arya because Tim was more confident in his presentation and had more relevant work experience on his CV. But actually Arya performed as well as Tim, and had just as much relevant work experience on her CV as Tim did. The director’s choice was driven by implicit biases against non-white (non-male/overweight) candidates. In this scenario, the director is not aware of the effects of implicit biases, and accounts for her choice on the basis of reasons that are not supported by the evidence at her disposal, such as the quality of the candidates’ performance during the interview or the relative strengths of their CVs.

The director’s claim can be easily regarded as an instance of confabulation. When explaining her decision, the director is not aware of some of the factors causally relevant to it (such as implicit biases), and provides reasons that are not well supported by the information she has. In the process of providing the explanation, she endorses other claims that lack evidential support and do not fit with the evidence made available in the CV and during the interview*,* such as “Arya is not as confident as Tim”, “Tim has more experience than Arya”, and so on. Just as in the incest case, not knowing which factors causally impact on her choice, the director offers reasons that are widely accepted as good reasons for hiring decisions.

The results of experiments on biased hiring decisions could be interpreted differently. For instance, the director may offer an explanation that involves no confabulation and is based on a false or poorly supported belief (“Tim has more experience than Arya”). Alternatively, she may offer a justification for her decision as opposed to an explanation of it, but the reasons she offers are epistemically problematic as reasons to prefer Tim over Arya because she has no evidence for Tim being more experienced or more confident than Arya.

I hope I have shown in this section that offering reasons for attitudes and choices leads to epistemic problems in a number of contexts and not just in consumer choice; and that confabulation in the sense I am discussing it here affects the non-clinical population as well as the clinical population with significant memory impairments. Next, I consider what the costs and benefits of non-clinical confabulation may be.

## Epistemic Costs of Confabulation

We saw that there are some interesting analogies between clinical and non-clinical confabulation. Table 1 offers a summary of such analogies.

**Table 1:** Analogies between clinical and non-clinical confabulation

Clinical confabulation	Everyday confabulation
The claim should be based on autobiographical information that is no longer available due to a memory impairment.	The explanation of a choice or attitude should be based on knowledge of factors that are opaque to introspection (e.g. priming effects, implicit bias).
The person making the claim is sincere and has no intention to deceive.	The person offering the explanation is sincere and has no intention to deceive.
The gap in knowledge is filled by a plausible claim.	The gap in knowledge is filled by a plausible explanation.

So, what features of an explanation make it an instance of non-clinical confabulation? I believe there are two necessary features and one optional feature that deserve attention.

Necessary features:

1. *Ignorance*: People ignore some of the key causal factors leading to the formation of their attitudes and choices.
2. *Ill-groundedness*: People produce ill-grounded claims about the causes of their attitudes and choices.

Common but optional feature:

3. *Further ill-groundedness*: As a result of producing the ill-grounded causal claim, people commit to further beliefs that, even if generally plausible, do not fit the specifics of the situation in which the attitude is formed or the choice is made.

When people confabulate they ignore some of the psychological processes responsible for the formation of their attitudes or the making of their choices, and produce an ill-grounded causal claim when asked for an explanation. The purpose of the rest of this section is to clarify what the epistemic costs of confabulation are and how my account relates to existing accounts of confabulation in the philosophical literature. In Section 5.1. I ask whether people’s ignorance of the causal history of their attitudes and choices has implications for self-knowledge intended as mental-state self-attribution. In Section 5.2. I consider why people offer ill-grounded explanations rather than acknowledging ignorance, and why they go on and commit themselves to further ill-grounded beliefs.

### Ignorance

The relevant philosophical literature suggests that confabulation is a failure of self-knowledge. For instance, on the basis of the evidence on pervasive confabulation about reasons for attitudes and choices, Lawlor (2003) argues that mental-state self-attributions lack authority as they are not as accurate as third-party attributions and fail to correlate with the person’s future behaviour. On similar grounds, Carruthers (2005) argues that there is no special first-personal route to self-knowledge. His influential view is that people attribute mental states to themselves in the same way as they attribute mental states to others, using interpretation.

On the basis that ill-grounded explanations of attitudes and choices are virtually indistinguishable from well-grounded ones and are very common, Scaife (2014, page 471) argues that we should be genuinely concerned about the reliability of self-knowledge. Thus, Strijbos and de Bruin (2015) are right in interpreting the standard philosophical account of confabulation as an instance of “failed mind-reading”: confabulation shows that people make mistakes in attributing mental states to themselves.

[If] confabulation turns out to be a widespread phenomenon in everyday social practice, this would seriously undermine first-person authority of mental state attribution. (Strijbos and de Bruin 2015, page 298)

Whether the form of non-clinical confabulation we are examining here involves a failure in mental-state self-attribution depends on what we take successful mental-state self-attributions to require. In their original paper on priming effects, Nisbett and Wilson are very clear that participants’ verbal reports are inaccurate because participants ignore the mental processes leading to their choices and, as a result, *misidentify* the reasons for their choices. Confabulation is evidence for the view that people are blind to the processes responsible for their choices, but does not imply that they are also blind to what choices they made. Independent of whether research participants can identify the reasons for their choices, their choices are *authentic*, in the sense that they are sincerely reported and genuinely endorsed. If successful mental-state self-attributions require awareness of one’s attitudes and choices, then they are not threatened by the form of confabulation reviewed here.^6^

Does successful mental-state self-attribution require that people are aware of the mental processes responsible for their attitudes and choices? This sounds like an implausibly demanding requirement. In the cases where confabulation has been observed and documented (such as consumer choice, moral judgements, and hiring decisions), causal factors leading to the attitude or the choice are likely to be psychological processes that involve priming effects, socially conditioned emotional reactions, and implicit biases whose role cannot be directly experienced or easily observed, but needs to be inferred on the basis of the systematic, scientific study of human behaviour.

Does successful mental-state self-attribution require that people’s subsequent behaviour is explained and reliably predicted on the basis of that self-attribution? This also sounds like an implausibly demanding requirement, one that imposes more stability and consistency on people’s mental life than is reasonable to expect. We do not know whether people who claim to have chosen a pair of stockings for its texture would choose the softest pair of stockings at their next consumer choice survey, but should they not do so, the fact that mental-state self-attributions fail to shape their future behaviour does not speak so much against self-knowledge as against the crystallization of preference criteria for stockings.

I have proposed here that the evidence of confabulation gathered in the literature on consumer choice and moral judgements and in the research on implicit biases in hiring decisions does not threaten self-knowledge as mental-state self-attribution. Research participants know the content of their attitudes and choices – they just ignore some of the mental processes contributing to them.

### Ill-Groundedness

We saw that when people confabulate they tell more than they can know, and offer ill-grounded causal claims as explanations for their attitudes and choices. In addition to that, people may also end up committing to beliefs that do not fit the specifics of the situation, as a result of producing ill-grounded causal claims.

It is not clear why people tell more than they can know. Processes of introspection, self-observation, or self-interpretation are not always reliable methods for identifying the causal factors responsible for attitudes and choices, and are vulnerable to error. So, when people are asked questions such as: “Why did you choose that nightgown?”, “Why do you believe that it was wrong for Julie and Mark to have sex?”, or “Why did you offer the job to Tim and not to Arya?”, most are not aware of the role of priming effects, basic emotional reactions, or implicit biases in their choices or attitudes. This is because such factors cannot be accessed via introspection, straight-forwardly observed, or inferred from behaviour, and thus cannot be easily identified. But if people do not know the reasons for their choices and attitudes, why don’t they just acknowledge ignorance?

People do not acknowledge their ignorance because *they do not know that they do not know* some of the key factors contributing to their attitudes and choices. In the accounts of confabulation developed by Hirsten (2005) and Coltheart and Turner (2009), people are not dishonest when they confabulate, but sincere, and convinced of the accuracy of their claims. When discussing the Nisbett and Wilson study, Coltheart and Turner argue that participants do not realise that they do not know the answers to the questions they are asked, and they accept as true the answers they provide (Coltheart and Turner 2009, page 185). This suggests that when people confabulate *they believe they know* how their attitudes and choices were formed, and this is due to the fact that information that would ground accurate explanations for their attitudes and choices is *unavailable* to them.

Information can be unavailable to a varying extent and for different reasons (see Sullivan-Bissett 2015 for details of the taxonomy I use here). We have a case of *strict* unavailability when the information that would ground the accurate explanation cannot be accessed or retrieved. If a person involved in a consumer survey is asked why she chose a particular pair of nylon stockings and does not know about priming effects, she lacks the information that would most likely ground the accurate explanation of her choice.

We have a case of *motivational* unavailability when there are motivational factors inhibiting the acceptance or use of the information that grounds the accurate explanation. The director of a company in charge of hiring decisions may become aware of the influence of implicit bias on people’s behaviour at an equal opportunities training workshop. Still, she may refuse to acknowledge that *she* is implicitly racist or sexist because this conflicts with her view of herself as an egalitarian. So, she continues to confabulate reasons for preferring male (non-overweight/white) candidates.

We have a case of *explanatory* unavailability when information that would ground the accurate explanation is not regarded as relevant to the target phenomenon, and thus is dismissed. The fact that people choose items due to their relative position may seem *outrageous* (as Nisbett and Wilson say in the passage I cited earlier), and thus the accurate explanation may be dismissed as implausible. Similarly, a person who is asked to explain why she believes that the incestuous relationship between Julie and Mark is wrong might have heard that people are socially conditioned to react with disgust to descriptions of incest. Yet, she might find it implausible that moral judgements are primarily determined by basic emotional reactions of disgust, insisting that her response was motivated by the endorsement of an ethical principle.

As we saw, when people provide an explanation for their attitudes and choices, their answers are based on general plausibility considerations about why stockings are chosen, incest is condemned, or a job candidate is selected. Because the answers are based on *general* plausibility considerations, they can be blind to *specific* features of the situation at hand. Although it is generally plausible that softness or brightness makes a pair of stockings preferable to another, it is false in the context of a choice between identical stockings that the chosen pair was softer or brighter. In the examples I considered, people commit to beliefs that do not fit the evidence such as: “The stockings on the right are more brightly coloured than those on the left”, “The siblings will be scarred by the experience of incest”, or “Tim was more confident than Arya”.

Couldn’t people offer an answer that fits the evidence better? People often do offer answers that are better supported by the evidence. Even if the answer remains an instance of confabulation, because it is not based on information relevant to the formation of the attitudes or the making of the choices, the confabulation is obviously less epistemically costly if it does not also commit the person to adopting further beliefs that are ill-grounded. Let me offer an example of an explanation that involves no further commitment to beliefs that do not fit the evidence.

Freya is asked to choose between two nightgowns that are not identical (this was one of the tasks in the original Nisbett and Wilson study). Let us assume that she chooses the nightgown on her right-hand side because it is on her right-hand side, but she is not aware of the role of position effects on her choice. When Freya is asked why she chose that nightgown she says that she chose it because it is softer. The nightgown she chose is indeed softer than the alternatives. In this case, Freya provides an inaccurate and ill-grounded explanation of her choice, as the explanation is not based on information relevant to why she made the choice. That said, the nightgown on her right-hand side *is* softer than the alternatives. Not knowing why she made that choice, and not knowing that she does not know, Freya provides a plausible explanation that does not commit her to any additional ill-grounded claims.

Similar scenarios can be constructed in the case of moral judgements or hiring decisions as well, and this suggests that instances of confabulation can be more or less epistemically costly depending on whether further ill-grounded beliefs are adopted.

## Benefits of Confabulation

In the previous section we saw that people confabulate when they ignore the causal processes responsible for their attitudes and choices and commit themselves to ill-grounded beliefs. Given our analysis so far, the prospects for such ill-grounded beliefs to have any benefits sound grim. However, in the empirical literature on clinical confabulation two sorts of benefits are discussed: *psychological adaptiveness*, which is usually characterised in terms of subjective wellbeing or good functioning; and *biological adaptiveness*, which is usually characterised in terms of genetic fitness.^7^ The two types of benefits do not always come together, as McKay and Dennett (2009) have observed. A belief or pattern of behaviour can be conducive to genetic fitness by increasing a person’s chances of survival and reproduction without being conducive to that person’s increased wellbeing or better psychological functioning, and viceversa. When we interact with our physical and social environment, there is also an epistemic dimension to our interactions that does not always receive a distinct acknowledgement in the psychological literature. That is the dimension I want to explore here. Can confabulation have *epistemic* benefits, broadly intended as positive effects on the acquisition, retention, and use of relevant information?

The empirical literature on dementia and amnesic syndromes suggests that some forms of confabulation can be psychologically adaptive, enhancing people’s wellbeing and and also helping people engage more fruitfully in rehabilitation programmes which improve their chances of recovery or adjustment (Fotopoulou 2008; Hydén and Örulv 2009; Weinstein 1996). One important aspect is that confabulation contributes to people’s sense of themselves as competent and largely coherent agents, enabling them to retain and share some key self-related information. An analogous claim can be made with respect to non-clinical confabulation.

I believe that the role of confabulation in what is often called ‘perceived agency’ or the ‘agentic self’ has both psychological and epistemic implications, where the potential epistemic benefits are sometimes mediated by the psychological ones. The person who sees herself in agentic terms tends to behave more like an agent and this often leads to better outcomes.

In facing these [life-course] challenges, an agentic individual is the primary origin of his or her actions, has high aspirations, perseveres in the face of obstacles, sees more and varied options, learns from failures, has a strong sense of well-being, and so on. (Little et al. 2006, page 63)

But let us consider how confabulation can make a contribution to perceived agency first. There are at least three relevant aspects of clinical confabulation that deserve attention here: (1) the construction of a better self; (2) the integration of self-related information; (3) the maintenance of the social self.

Confabulating can enhance the person’s wellbeing when the content of the confabulation presents the person in a better light than is the case. For instance, in the confabulation the person’s independence, talents, or competencies may be exaggerated. Laura who has dementia claims that she was working in the office this morning but she was actually being cared for in hospital. Her inaccurate report conjures an image of herself as healthy, self-sufficient, and industrious. But Laura’s actual situation is very different: Laura has been retired for some time and she has lost her independence due to the advanced stage of her debilitating illness.^8^ Her report makes reference to her pre-morbid self rather than her current self. When people report a memory that is distorted because it presents them as more independent, talented, or competent than they actually are, the distortion often contains some key information about their autobiographical past, and makes them feel better about themselves supporting the belief that they are successful in some specific, valued context.^9^

Further, by confabulating people integrate self-related information into a largely coherent body of knowledge that helps them make sense of the situation in which they find themselves. Recall the example of Henry who does not remember all the details of his married life, but answers questions about it nonetheless, striving to tell a coherent story. Henry reconciles the fact that he remembers having been married for 4 months with the fact that he knows he has four children by claiming that his children were adopted, Instead, he had four children from his wife in over 30 years of marriage. Although it is false that he had been married for just 4 months and that his children were adopted, confabulating allows him to impose some coherence on the fragmented and often conflicting information that he still possesses about himself.^10^

Related to the previous two aspects, confabulating helps “establish and maintain a personal identity in interactions with others” (Hydén and Örulv 2009, page 25). When autobiographical memory is compromised, there are fewer opportunities to verbalise and share self-related information. People with dementia or amnesia may feel less confident about social exchanges and fear sanction if inaccuracies in their reports are detected. When they are assailed by self-doubt or experience external challenges, they tend to withdraw from social interactions. Persevering in reporting autobiographical facts is beneficial in this context, even when reports turn out to be repetitive, distorted, or incomplete, because it enables people to maintain some exchanges with their peers and consolidate the accurate information they still have about themselves.

There is some obvious overlap among the three benefits identified in the literature on clinical confabulation, and all three are primarily psychological: the construction of a better self leads to greater subjective wellbeing and supports self-esteem at a time when this is threatened; the integration of self-related information leads to a more coherent self-image and self-narrative, avoiding the tension caused by conflicting information; and the maintenance of a social self enables information exchanges and feedback from peers, reducing the risks of withdrawal and isolation.

Further, the three roles we identified can be said to contribute to people’s sense of themselves as competent and coherent agents, which is challenged by severe memory impairments. Arguably the importance of preserving an agentic self is not merely psychological, and epistemic benefits can also ensue, such as the opportunity to preserve and share key self-related information (Bortolotti and Sullivan-Bissett forthcoming). Despite the loss of autobiographical memory, people who confabulate continue to exchange information and to see themselves as the experts in the subject-matter that is their own life. The preservation and consolidation of self-defining beliefs are epistemic benefits, not mediated by psychological benefits.

Moreover, when people construct a better self they avoid negative feelings of disorientation and incompetence that could become overwhelming. The successful management of overwhelming negative emotions has consequences for the capacity to relate to others, and to interact with the surrounding physical and social environment. An active engagement with the world is also an epistemic goal, mediated this time by psychological benefits (such as not being consumed by overwhelming negative emotions). Especially in the context of dementia, confabulation counteracts the negative effects of social isolation on the capacity to “express and explore identity” (Bouchard Ryan et al. 2009, page 145). By filling gaps in knowledge about the past, confabulations support the level of communication required for meaningful social interactions, helping preserve the capacity and willingness to exchange information with other people and receive feedback from them (Small et al. 1998, page 291; Hydén and Örulv 2009; Addis and Tippett 2004). This leads to another epistemic benefit: when people have the opportunity to share information, they can also be challenged about what they share, they build more critical distance from their reports, and some of their inaccurate beliefs become less rigid and less entrenched as a result. Although socialisation is a psychological benefit, exchanging information and obtaining feedback are central epistemic goals, whose positive consequences include the acquisition of new true beliefs and the correction of existing false beliefs.

Can non-clinical confabulation have analogous benefits?

## Everyday Confabulation and Perceived Agency

The costs and benefits of everyday confabulation are less evident than those of clinical confabulation, because everyday explanations of attitudes and choices are not responses to a breakdown, but “fixes” to a form of ignorance that can be remedied by learning about the causal factors responsible for people’s attitudes and choices. That said, both everyday and clinical confabulations involve ill-grounded beliefs, and both can play a useful role in supporting a person’s sense of herself as a competent and largely coherent agent.

As clinical confabulation, so non-clinical confabulation contributes to the *construction of a better self*. In the everyday context, people self-enhance by seeing themselves as competent agents and decision makers who do and believe things for (good) reasons as opposed to people whose attitudes and choices are randomly determined by external cues or unconscious drives. When a request for an explanation is made and the accurate explanation is not available, offering an explanation that is articulate and plausible is preferable to replying “I don’t know” from this point of view. Coltheart (2017) argues that confabulation as a general phenomenon can be seen as an example of the *drive for causal understanding* studied by Alison Gopnik (2000). At a mere unconscious level, the drive motivates people to develop theories for the phenomena they do not yet understand. Often such theories pick out veridical maps of causal relations among phenomena and are straight-forwardly adaptive in a biological and epistemic sense, but some of the time they get the causal relations wrong. Confabulations are such a case. On the one hand, ill-grounded explanations can be easily shared and support the person’s sense of herself as a competent agent and decision maker, that is, someone who can tell what the reasons for her choices are. On the other hand, ll-groundedexplanations often misidentify the causal relationships between a person's reason and her choice or attitude.

Non-clinical confabulations can also help identify threads in the person’s attitudes and choices. Such threads make the person’s overall commitments more meaningful to herself and others. Recall our previous examples: the company director will see herself as someone who values self-confidence in her employees and relevant work experience in job candidates; the person asked to judge a case of incest between siblings will see herself as someone who morally disapproves of situations that typically cause harm to the people involved. Thus, non-clinical confabulation can also play the role of *integrating self-related information*. In spite of being ill-grounded, explanations for attitudes and choices help embed individual attitudes and choices in a more comprehensive narrative, where reasons form general patterns that the person uses to make sense of her past behaviour and to predict and even direct her future behaviour. In particular, in the cases we considered here, confabulation enables a person to integrate an instance of behaviour whose causes are at least partially mysterious (opaque to introspection or difficult to infer) into a wider system of beliefs, preferences, and values that contributes to her overall self-image.

Attitudes and choices are often malleable and unpredictable, and strongly dependent on contextual cues, as the Nisbett and Wilson study, the Schnall et al. (2008) study, and many other studies in a variety of research programmes show. However, it is common for agents to deny fluctuation and impose some stability and coherence on their own behaviour. Preferences may vary considerably depending on the circumstances, and this is also due to such preferences being influenced by mental processes that cannot be controlled via deliberation or accessed via introspection, such as priming effects, basic emotional reactions, and implicit biases. In spite of this variation, people tend to see their preferences as stable and consistent across time and across contexts, and maintain this illusory consistency by confabulating.

The illusion of consistency often leads to better psychological and pragmatic outcomes than the more accurate acknowledgement of fluctuation. For instance, in a job search graduates who downplay the inconsistency of their preferences are more likely to feel good about themselves and their prospects, and to succeed in obtaining desirable job offers, than those who have a more realistic view of their own fluctuating preferences (Wells and Iyengar 2005). Thus, one of the benefits of confabulation is that it enables people to develop threads joining their experiences together and to present themselves as largely coherent, lowering the anxiety that comes with self-doubt by over-emphasising integration. This leads to an increased sense of self-efficacy which serves to sustain the motivation to pursue goals in the face of difficulties (Bandura 1989).

As with clinical confabulation, also non-clinical confabulation plays an obvious *maintenance of the social self* role by enabling information to be shared. Thalia Wheatley argues that assigning meaning to behaviour helps develop social connections: “[t]he healthy human brain is not a veridical recorder of events but rather a *meaning machine* that fills in gaps, rearranges time and space, delays conscious experience, and generates false explanations via available cultural theories” (Wheatley 2009, page 218, my emphasis). The view that the brain does not aim at *accuracy* but at *coherence* is often interpreted in a simplistic way, as proposing a straight-forward trade-off between psychological and epistemic goods, where accuracy is exchanged for whatever is needed to attain a significant increase in wellbeing or some psychological adjustment. However, seeing oneself as a competent and largely coherent agent has positive epistemic consequences.

Ill-grounded explanations for attitudes and choices allow a conversation about those attitudes and choices to develop, among peers and within oneself, promoting external feedback or personal reflection on the issues that are relevant to the formation of those attitudes or the making of those choices. Bertram Malle (2004) argues that when people confabulate after being asked to explain their behaviour, interpersonal communication is facilitated. Hugo Mercier (2011) claims that offering arguments to explain judgements or behaviour play an important function especially at group level, where the argument can be shared, evaluated, and corrected. If the judgement or behaviour were recognised as something that requires no explanation, or that has been arrived at by a random process akin to guessing, the person would lose the sense of agency and efficacy that comes from viewing the attitude or choice as something that reflects her beliefs, preferences, and values.

In the context of choosing nightgowns and stockings, it may not be so important to appear as a competent agent and decision maker who is aware of the reasons for her choices and chooses items for good reasons. However, the implications of an attitude or choice on perceived agency become more significant when the attempted explanation concerns moral attitudes or hiring decisions, which the person identifies with and which can be relied on to shape the person’s future behaviour. Articulating reasons for *self-defining* attitudes and choices can be a starting point for dialogue and reflection, potentially leading to self-criticism and self-improvement.

By contributing to self-enhancement and integration of self-related information and by playing a social role, everyday confabulation supports people’s perceived agency. When perceived agency is strong, and people feel that they have the capacity to pursue and fulfil their goals, then they act more like agents and their motivation is sustained in critical circumstances. Psychological research has shown that people who self-enhance are not only more likely to persist in pursuing their goals in the face of set-backs, but, at least in some domains, they are also more likely to perform satisfactorily and fulfil their goals. They tend to be more productive, more resilient, better at planning, and more effective at problem-solving (e.g., Alicke and Sedikides 2009; Hepper and Sedikides 2012). Psychological research on self-efficacy and self-determination have also established that people who view their attitudes and choices as driven by reasons, and whose attitudes and choices are integrated in a coherent pattern of behaviour, are more likely to pursue and fulfil their goals (Bandura 1989; Deci and Ryan 1985).

So far I suggested that a plausible but ill-grounded explanation may be better than no explanation at all for the purposes of allowing people to share information about their attitudes and choices, and elicit feedback from others.^11^ But surely my opponent would argue that a plausible and *well-grounded* explanation, including the *accurate* explanation, would be far more advantageous, at least from an epistemic point of view. Well-grounded explanations fare better than ill-grounded ones at representing reality accurately, by tracking the correct causal relationships, and enabling understanding – for instance, an understanding of the factors influencing one’s choices. This is a fair point, and indeed the benefits of non-clinical confabulation I have discussed do not neutralise its evident epistemic costs.

However, a sophisticated analysis of the epistemic status of confabulation needs to take into account both costs and benefits. A better-grounded or even accurate explanation, such as the explanation research participants in the Nisbett and Wilson study may be offering after debriefing (“My choice of this pair of stockings must be due to position effects I was not aware of”), is unlikely to play self-enhancing and self-integrating roles to the same extent as the rival explanation (“I chose this pair of stocking because it is softer”). Acknowledging that the consumer choice was not based on the quality of the items but on an unconscious tendency to favour items on the right-hand side may not support people’s sense that they are competent agents and decision makers, and may not help them identify patterns that contribute to their construction of a coherent image of themselves as discerning consumers.

Thus, the confabulation fares worse than the accurate explanation at representing reality accurately and promoting a well-rounded understanding of people’s behaviour, but fares better than the accurate explanation at supporting the person’s perceived agency. Also notice that, in many of the circumstances we discussed the comparison between offering a well-grounded explanation and an ill-grounded one is merely a theoretical possibility. This is because the information relevant to the formation of the attitude or the making of the choices either is not available, or can only be attained by learning about the role priming effects, basic emotional reactions, or implicit biases on attitude formation and decision making. If steps could be taken to make the relevant information more widely available, thereby enabling people to provide better-grounded explanations, then the question would be how to preserve some of the beneficial effects of confabulation. I will come back to this in the next section.

## Objections and Implications

I argued that ill-grounded explanations for attitudes and choices support people’s sense of themselves as competent and largely coherent agents, thereby sustaining their motivation to pursue their goals, and enable socialisation, thereby affording the opportunity to reflect and receive feedback on the reasons for said attitudes and choices. Although I characterised the overall positive contribution of confabulation as a contribution to perceived agency, some of the benefits I discussed rest on the opportunity for the attainment of epistemic goals, such as self-correction and self-improvement, and thus seem to be distinctly epistemic. In Table 2 I summarise the main benefits of everyday confabulation, comparing them with those of clinical confabulation.

**Table 2:** Summary of the benefits of clinical and everyday confabulation

Clinical confabulation	Everyday confabulation
Construction of a more independent, talented, and competent self.	Perception of oneself as a competent agent who believes and does things for good reasons.
Integration of self-related information in a coherent narrative.	Perception of oneself as a largely coherent agent who has a stable set of beliefs, preferences, and values.
Maintenance of a social self able to share self-related information.	Participation in exchanges of information, facilitating personal reflection and peer feedback.

But the view that confabulations may have epistemic as well as psychological benefits raises several concerns, and it is important to think about its implications.

- *(1) Does confabulation really promote socialisation and enable feedback that would not be forthcoming otherwise?*

One concern is that providing misleading information will in time discourage people from exchanging information with the confabulator and compromise (rather than promote) socialisation. In other words, the confabulator whose explanations are recognised as ill-grounded may be socially sanctioned and excluded from future exchanges, or her contributions to the information exchange may be dismissed as untrustworthy. This point is particularly relevant in cases of clinical confabulation, when the report may be evidently false as in the examples of Henry and Laura we discussed earlier. It is less of a concern in cases of non-clinical confabulation, when the ill-grounded explanation is often plausible (see Milhailov 2016; Coltheart 2017) and sometimes feels more intuitively right than the accurate explanation. A key factor here seems to be whether the confabulation gives rise to further ill-grounded beliefs that can be more easily exposed as ill-grounded, because the evidence relative to those further beliefs is likely to be more directly available to the confabulator and her peers. An ill-grounded explanation in the non-clinical context that does not commit the confabulator to further ill-grounded beliefs is likely to support rather than undermine socialisation.

Another concern is that providing an answer to the request for explanation may close off the conversation more than acknowledging ignorance because it gives the false impression that the confabulator already knows what she is talking about. Wouldn’t a more open answer such as “I don’t know” or “I’m not sure” elicit more constructive feedback from the agent’s peers? I believe that this depends on what an “I don’t know” or an “I’m not sure” answer would be taken to mean. If it were interpreted as: “I think this question has an answer but I don’t know what the answer is”, then it would be as conducive to debate as the confabulation, and even more apt at promoting an exchange of views between the person and her peers. If it were interpreted as something like: “This choice has no reason” or “I chose randomly”, then it would seem to close off further speculation as it would suggest that the choice was entirely out of the agent’s conscious and deliberative control. Further, notice that an “I don’t know” or an “I’m not sure” answer would not contribute to perceived agency to the same extent as the ill-grounded explanation, as it would not enable the person to see herself as a competent decision maker who chooses for good reasons.

Finally, are the benefits discussed in Section 6 and 7 genuinely epistemic? Arguably, the opportunity to share information and receive feedback is an epistemic gain that is not mediated by an increase in wellbeing (although socialisation also has independent psychological benefits). When a belief is challenged, the situation can be psychologically distressing in clinical contexts where the person’s assertiveness and self-esteem may be already seriously undermined by adverse circumstances. But in the context of non-clinical confabulation, negative feedback enables people to think further about their explanation and consider the possibility that it should be revised or rejected (see Mercier 2011 for a similar point). In addition, the challenge can focus people’s attention on reasons for the reported choice or attitude that they would not have considered in the absence of the challenge. Thus, socialisation with its opportunities for exchange of information and external feedback seems to offer the opportunity to reap some epistemic benefits that are independent of the psychological benefits of confabulation.

- *(2) If the sense of competence and coherence the agent gets from the confabulation is illusory, how can they have epistemic benefits?*

Part of the reason why the philosophical literature has been silent about the potential epistemic benefits of confabulation is that there is a strong resistance to accepting the idea that a false or illusory belief can lead to the fulfilment of epistemic goals. Can we ever get epistemic benefits from a false or illusory belief? A belief that makes us explore a certain subject matter further may be inaccurate and still play an important heuristic role, enabling us to gain new accurate information. One example is the discussion of “useful fictions” in the philosophy of science (e.g., Suárez 2013).

In the present case, if I am right, the tendency to offer ill-grounded explanations makes people feel and behave more like agents, contributing to attainment of some of their goals, including their epistemic goals. If people see themselves as agents who believe and choose for good reasons and are moved by plausible considerations, they can be more likely in the future to believe and choose for good reasons and be moved by plausible considerations when they have a more active role to play in the formation of their attitudes and the making of their choices. This point is well made by Strijbos and de Bruin (2015) who focus on the future-oriented, mind-shaping aspect of self-ascriptions.

- *(3) Why does it matter whether confabulation supports perceived agency or has epistemic benefits?*

Given that ill-grounded explanations for attitudes and choices are epistemically costly by leading to further ill-grounded beliefs and preventing a fuller understanding of the reasons for people’s attitudes and choices, measures should be taken to eliminate or reduce the amount of confabulation in everyday explanations. For instance, one proposal would be to make information that could ground accurate explanations more readily available by, say, teaching schoolchildren what the role of priming effects, basic emotional reactions, and implicit bias can be in attitude formation and decision making. If it could be demonstrated that this kind of intervention contributes to reducing confabulation by making the accurate explanations more salient and less ‘implausible’, then it sounds like it should be implemented. The possibility that confabulation has some benefits, though, and benefits that better-grounded explanations may lack, suggests that we should also think carefully about what could replace ill-grounded explanations in their more positive role, that is, in supporting the sense of oneself as a competent and largely coherent agent.

One possibility is to devise strategies that can be used to override or compensate those influences on attitude formation and decision making that do not necessarily reflect a person’s beliefs, preferences, and values, and that may lead to biased judgements (priming effects, socially conditions basic emotional reactions, implicit biases). An acknowledgement that people can retain the capacity to vindicate their attitudes and choices as *actively deliberated* rather than as *largely determined by external cues* would increase people’s sense of agency and alert them to their general tendency to fill gaps and confabulate.

## Conclusions

After describing everyday confabulation and illustrating the phenomenon with some examples, I proposed that this form of confabulation has two epistemic costs. First, the main causal claim offered as an explanation is ill-grounded due to ignorance of some of the causal factors contributing to forming attitudes and making choices. Second, confabulating may also lead one to commit to further ill-grounded beliefs.

Next, I argued that confabulation has some benefits as well as costs. Given that typically the accurate explanation is unavailable due to cognitive limitations, motivational factors, or explanatory constraints, dumbfounding would be the only alternative to confabulating and would prevent people from making sense of their own behaviour as motivated by reasons. Ill-grounded explanations fill gaps in knowledge, and, thanks to their self-enhancing and self-integrating roles, make a contribution to people’s sense of themselves as competent and largely coherent agents.

As a result, confabulation makes it easier for people to preserve their motivation to pursue their goals, increasing the chance that they fulfill some of their goals, including their epistemic goals. Confabulation also enhances socialisation which gives people the opportunity to verbalise and share both self-related information and reasons for attitudes and choices.

There is no denying that confabulation has epistemic costs. However, in some circumstances, the positive contribution of ill-grounded explanations to perceived agency can translate into epistemic advantages. One is that the explanation can be the starting point for constructive exchanges with peers and for further reflection on one’s attitudes and choices.

Finally, I considered some implications of the epistemic benefits of confabulation. In devising measures to reduce everyday confabulation we should also think about how else to support positive self-construction, successful integration of self-related information, and socialisation.

## Footnotes

1 This example is inspired by a real-life case discussed by Moscovitch (1995).

2 In-depth analyses of this study are provided in Hirsten (2005) and Carruthers (2005), and more recently Strijbos and de Bruin (2015), Milhailov (2016) and Coltheart (2017).

3 In a recent paper, Newell and Shanks (2014) argue against the standard interpretation of the Nisbett and Wilson study, hypothesising that research participants’ choices were not determined by the position of the items. I do not believe the new interpretation would make the present discussion outdated or irrelevant, as one could agree with Newell and Shanks about what process is responsible for bringing about the participants’ choices, and yet consider the results of the Nisbett and Wilson study as evidence for confabulation.

4 See for instance: Pingitore et al. (1994), Lieber (2009), Steinpreis et al. (1999), Agerström (2014), and Agerström and Rooth (2011).

5 See for instance: Holroyd (2012), and Sullivan-Bissett (2015).

6 Some arguments for the view that the form of confabulation examined in this paper does not threaten mental-state self-attribution can be found in Bortolotti (2009). One may argue that mental-state self-attribution is threatened by other forms of confabulation, such as choice blindness. An in-depth analysis of this claim is outside the scope of the present paper, but it will suffice to say that there are interpretations of the choice blindness phenomenon which are incompatible with this claim (see for instance Lopes 2014).

7 Psychological and biological adaptiveness do not exhaust the kinds of benefits that cognitions can have, but are the most commonly referred to in discussions of confabulation.

8 This example is inspired by a case discussed in Hirstein (2009).

9 For an excellent discussion of this point, please see Fotopoulou (2008, page 555).

10 See Moscovitch (1995, page 229) for a description of this aspect of confabulation.

11 An utterly implausible explanation may not support social functioning if it makes the person appear untrustworthy as a source of information.
